# The effect of a change in fluid intake advice on outcomes in chronic heart failure: a prespecified subanalysis of the FRESH-UP study

**DOI:** 10.1093/eschf/xvag197

**Published:** 2026-07-29

**Authors:** Job J Herrmann, Hans-Peter Brunner-La Rocca, Lisette E H J M Baltussen, Fabienne Beckers-Wesche, Sebastiaan C A M Bekkers, Louise Bellersen, J W Martijn Van Eck, H Carlijne Hassing, Tiny Jaarsma, Gerard C M Linssen, Ron Pisters, Sandra Sanders-Van Wijk, Marjolein H I Verdijk, Laura Rodwell, Roland R J van Kimmenade, D H Frank Gommans

**Affiliations:** Department of Cardiology, Radboud University Medical Centre, Geert Grooteplein 10, P.O. Box 9101, Nijmegen, 6525 GA, The Netherlands; Netherlands Heart Institute, Utrecht, The Netherlands; Department of Cardiology, Maastricht University Medical Center, Maastricht, The Netherlands; Cardiovascular Research Institute Maastricht (CARIM), Maastricht University, Maastricht, The Netherlands; Department of Cardiology, Radboud University Medical Centre, Geert Grooteplein 10, P.O. Box 9101, Nijmegen, 6525 GA, The Netherlands; Department of Cardiology, Maastricht University Medical Center, Maastricht, The Netherlands; Department of Cardiology, Bernhoven Hospital, Uden, The Netherlands; Department of Cardiology, Radboud University Medical Centre, Geert Grooteplein 10, P.O. Box 9101, Nijmegen, 6525 GA, The Netherlands; Department of Cardiology, Jeroen Bosch Hospital, ‘s-Hertogenbosch, The Netherlands; Department of Cardiology, Radboud University Medical Centre, Geert Grooteplein 10, P.O. Box 9101, Nijmegen, 6525 GA, The Netherlands; Department of Health, Medicine and Caring Sciences, Faculty of Medical and Health, Linköping University, Linköping, Sweden; Department of Cardiology, University Medical Center Utrecht, Utrecht, The Netherlands; Department of Cardiology, Hospital Group Twente, Almelo/Hengelo, The Netherlands; Department of Cardiology, Rijnstate Hospital, Arnhem, The Netherlands; Department of Cardiology, Zuyderland Medical Center, Henri Dunantstraat 5, Heerlen, 6419 PC, The Netherlands; Department of Cardiology, Radboud University Medical Centre, Geert Grooteplein 10, P.O. Box 9101, Nijmegen, 6525 GA, The Netherlands; IQ Health Department, Radboud University Medical Center, Nijmegen, The Netherlands; Department of Cardiology, Radboud University Medical Centre, Geert Grooteplein 10, P.O. Box 9101, Nijmegen, 6525 GA, The Netherlands; Department of Cardiology, Zuyderland Medical Center, Henri Dunantstraat 5, Heerlen, 6419 PC, The Netherlands; Department of Cardiology, Radboud University Medical Centre, Geert Grooteplein 10, P.O. Box 9101, Nijmegen, 6525 GA, The Netherlands; Department of Cardiology, Máxima Medical Center, Veldhoven, The Netherlands

**Keywords:** Heart failure, Fluid restriction, Liberal fluid intake, Health status, Thirst, Lifestyle

## Abstract

**Background and Aims:**

The Fluid REStriction in Heart failure versus liberal fluid UPtake (FRESH-UP) study (NCT04551729) randomized patients with chronic heart failure (HF) to liberal fluid intake (LFI) vs fluid restriction (FR). Pre-trial fluid management may have impacted trial outcomes.

**Methods:**

The open-label FRESH-UP study randomized 504 patients with chronic HF to an advice of LFI or FR of 1500 ml/day. Primary outcome was the Kansas City Cardiomyopathy Questionnaire Overall Summary Score (KCCQ-OSS) after 3 months adjusted for baseline. Key secondary outcome was thirst distress. Patient-reported fluid intake was assessed at Week 6. Safety outcomes included death and hospitalizations. For this subanalysis, participants were stratified according to pre-trial fluid management (FR or LFI).

**Results:**

Prior to randomization, 269 (53.4%) patients adhered to FR and 235 (46.6%) adhered to LFI. After 3 month follow-up, KCCQ-OSS was significantly different between groups (*P* = .035): in patients changing from pre-trial FR to LFI, KCCQ-OSS was 74.7 [95% confidence interval (CI) 71.2–78.3] compared to 70.2 (95% CI 66.3–74.2) in those with the reversed scenario. In the latter group, thirst distress increased the most. Patient-reported fluid intake was significantly higher in patients randomized to LFI when compared to FR (*P* = .019), regardless of pre-trial FR or LFI (1771 and 1757 ml, vs 1486 and 1464 ml, respectively). Safety outcomes did not differ.

**Conclusions:**

These analyses add understanding to the effects of fluid intake management in chronic HF and expose a signal of potential harm of FR in patients with pre-trial LFI and a potential benefit of LFI in patients with pre-trial FR. These results corroborate with the primary results and support that there is no need to routinely initiate or continue FR in stable patients with chronic HF.

## Graphical Abstract

**Graphical Abstract:**
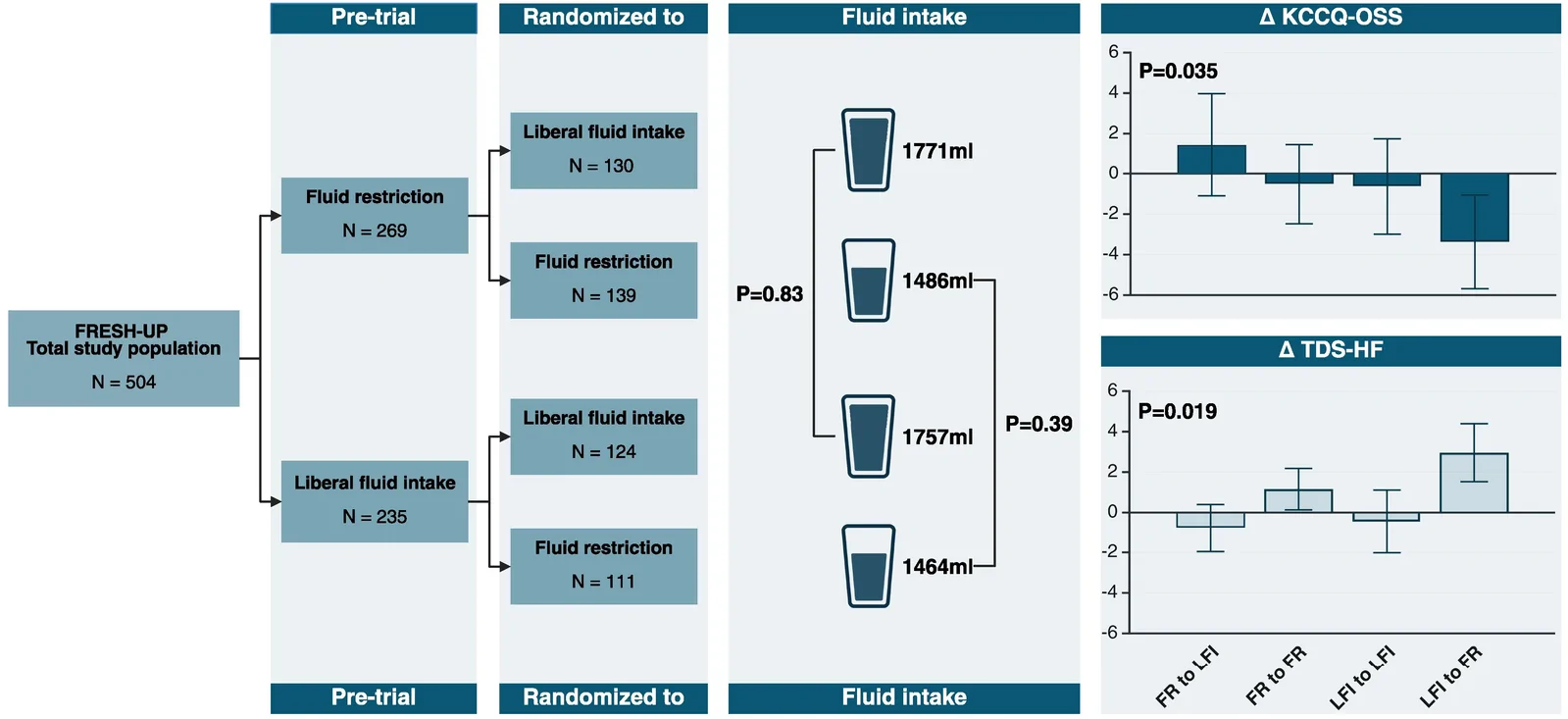

The effect of a change in fluid intake advice on outcomes in chronic heart failure. FR, fluid restriction; HF, heart failure; KCCQ-OSS, Kansas City Cardiomyopathy Questionnaire Overall Summary Score; LFI, liberal fluid intake; TDS-HF, Thirst Distress Scale for patients with HF.

## Introduction

Fluid restriction (FR) is frequently advised in daily clinical practice to patients with chronic heart failure (HF) in order to prevent congestion. But, evidence from clinical trials to support this advice is lacking.^1–5^

The Fluid REStriction in Heart failure versus liberal fluid UPtake (FRESH-UP) study is the largest clinical trial in this field.^1–5^ In this study, patients with symptomatic HF who were randomized to liberal fluid intake (LFI) had a higher health status, as assessed by the Kansas City Cardiomyopathy Questionnaire (KCCQ), after 3 months than those who were advised to restrict their fluid intake (*P* = .06).^1^ In the unrestricted patients, thirst distress was significantly lower, in the absence of a signal of harm related to LFI.^1^

In light of these findings, it can be put forward that FR should no longer be routinely recommended to patients with chronic HF.^1^

However, given the longstanding tradition of FR in HF, patients as well as clinicians may be reluctant to drop FR and adopt a liberal intake. This reluctance may be fed by the fact that little is known about the effects of changes in fluid management and that the difference in daily fluid intake between randomization groups was only about 280 ml. Fortunately, the FRESH-UP study provides a unique opportunity to study the effects of a change in fluid management in a well-described cohort of chronic HF patients, because *prior to randomization*, about half of the study population adhered to FR and the other half had a normal LFI. Accordingly, the present prespecified subanalysis focusses on the effects of pre-participation fluid management, and especially a *switch* in fluid intake, on health status, thirst distress, daily fluid intake, and safety outcomes. The results of this more in-depth analysis may have direct implications on the clinical question whether FR may be safely abandoned in patients with chronic HF.

## Methods

This is a prespecified subanalysis of the FRESH-UP study (NCT04551729). Patients were stratified based on their self-reported fluid management before study participation. Patients were categorized as pre-trial FR, if they self-reported adhering to some form of FR before study participation and therefore perceived themselves as ‘restricted’ regardless of actual fluid intake. Patients were categorized as pre-trial LFI, if they did not restrict their fluid intake before study participation.

The FRESH-UP study is an investigator-initiated, multicentre, randomized, open-label clinical trial, which evaluated the effect of an advice of LFI vs an advice of FR up to 1500 ml per day for a period of 3 months. The trial was approved by the Medical Research Ethics Committee of Radboud University Medical Center and all institutional review boards of the participating sites. All patients provided written informed consent before participation. The design and primary results have been previously published.^1,6^

Briefly, 504 outpatient adult patients with chronic HF with New York Heart Association (NYHA) class II or III, regardless of left ventricular ejection fraction, were included from May 2021 to June 2024 in the Netherlands. Key exclusion criteria were HF hospitalization, coronary intervention or implantation of a pacemaker device 3 months prior to randomization, changes in medical HF therapy in the 14 days prior to randomization, a glomerular filtration rate <30 ml/min/1.73 m^2^, and hyponatremia (defined as a sodium concentration <130 mmol/l). A full list of exclusion criteria is provided in the design paper.^6^

The primary outcome was health status as assessed by the KCCQ Overall Summary Score (KCCQ-OSS) at 3 months after randomization. Thirst distress as assessed by the Thirst Distress Scale for patients with Heart Failure (TDS-HF) at the same timepoint was the key secondary outcome. Additional outcomes assessed were KCCQ subdomains, proportion of patients with differences of 5 points or more in KCCQ-OSS compared to baseline, European Quality of Life Five Dimensions Five Levels questionnaire (EQ-5D-5L), patient-reported fluid intake at Week 6, and safety outcomes (e.g. safety events: death, all-cause and HF hospitalization, the requirement of intravenous loop diuretics and acute kidney injury during the total 6 months of clinical follow-up, and changes in N-terminal pro–B-type natriuretic peptide concentrations and weight).

### Study procedures

At baseline before randomization and at 3 months post-randomization, the KCCQ, TDS-HF, and EQ-5D-5L were completed. Six weeks after randomization, patients filled out a fluid-intake diary for 7 sequential days. Patients were clinically evaluated according to standard clinical practice, and adverse events were assessed during the visits at 3 and 6 months post-randomization. Fluid management after Month 3 was at the discretion of the treating physician and the patient. Study participation ended after the 6 month visit.

### Statistical analysis

Patient characteristics were summarized as numbers and percentages, means and standard deviations, or medians and interquartile ranges, whichever appropriate.

For this subanalysis, patients were stratified based on their self-reported fluid management before study participation (i.e. pre-trial FR or pre-trial LFI).

The difference in KCCQ-OSS and TDS-HF after 3 months was tested with analysis of covariance (ANCOVA), using baseline KCCQ-OSS and TDS-HF, respectively, as a covariate. Baseline fluid management was included as an interaction term, and significant baseline differences were added as covariates in the ANCOVA model to assess effect modification. Regardless of the outcome of this interaction analysis, as a nonsignificant result does not indicate homogeneity of randomization effects across the subgroups, the current subanalysis on change in fluid management was prespecified in the statistical analysis plan and as such performed to explore the potential effects of a change in fluid management. Clinical and safety outcomes were compared between randomization arms, stratified by pre-trial fluid management (i.e. pre-trial adherence to FR or LFI, and randomization to an advice of LFI or FR). Analyses of the other secondary continuous patient-reported outcome measures followed a similar approach for the primary analysis (i.e. other KCCQ summary scores and EQ-5D-5L). Between-group differences in proportions and continuous outcomes were analysed with χ^2^ test and Kruskal Wallis test, respectively. Patient-reported fluid intake was compared within each randomization arm and stratified by pre-trial fluid management. Continuous data on fluid intake were analysed using the Mann–Whitney U test, and between-group differences in proportions of patients within each randomization arm were analysed using the χ^2^ test. A *P*-value of .05 was considered significant. Analyses were performed with SPSS software version 29 (IBM).

## Results

### Patient characteristics

Baseline characteristics of the patients according to the pre-trial fluid management are shown in *Table 1*. A total of 269 (53.4%) patients adhered to some form of FR prior to study participation. Patients who adhered to a pre-trial FR were younger (67.8 ± 11.3 vs 70.8 ± 9.8), less frequently male [168 (62.5%) vs 171 (72.8%)], were prescribed more β-blockers [247 (91.8%) vs 203 (86.4%)] and used more frequently loop diuretics [151 (56.1%) vs 107 (45.5%)]. KCCQ-OSS and TDS-HF did not differ between patients adhering to FR or LFI before trial participation.

**Table 1:** Characteristics of the patients at baseline

	Pre-trial FR (*N* = 269)	Pre-trial liberal fluid intake (*N* = 235)	*P* value
Age, years	67.8 ± 11.3	70.8 ± 9.8	.001
Male	168 (62.5%)	171 (72.8%)	.01
White^a^	265 (98.5%)	227 (96.6%)	.22
**Quality of life**			
KCCQ-OSS	76.8 [61.5–89.2]	77.6 [59.9–88.5]	.86
TDS-HF	16.0 [11.0–23.0]	15.0 [10.0–20.0]	.15
EQ-5D-5L	0.82 [0.72–0.92]	0.82 [0.71–0.90]	.46
**NYHA functional class**			.13
II	240 (89.2%)	199 (84.7%)	
III	29 (10.8%)	36 (15.3%)	
**LVEF**			
%	39.5 ± 11.1	41.1 ± 10.5	.10
HFrEF	140 (52.0%)	120 (51.1%)	.82
HFmrEF	71 (26.4%)	59 (25.1%)	
HFpEF	58 (21.6%)	56 (23.8%)	
**Cause of HFe**			.21
Ischaemic	111 (41.3%)	110 (46.8%)	
Non-ischaemic	158 (58.7%)	125 (53.2%)	
**HF duration, years**	4.0 [1.0–10.0]	5.0 [2.0–10.0]	.21
**Fluid management pre-study participation**			<.001
FR up to 1000 ml	3 (1.1%)	0 (0.0%)	
FR up to 1500 ml	89 (33.1%)	0 (0.0%)	
FR up to 1500 to 2000 ml	147 (54.6%)	0 (0.0%)	
FR up to 2000 ml	30 (11.2%)	0 (0.0%)	
Liberal fluid intake	0 (0.0%)	235 (100%)	
**HF treatment**			
RAASi	251 (93.3%)	221 (94.0%)	.74
ACEi	42 (15.6%)	35 (14.9%)	.82
ARB	39 (14.5%)	39 (16.6%)	.52
ARNI	170 (63.2%)	149 (63.4%)	.96
β-blocker	247 (91.8%)	203 (86.4%)	.049
MRA	222 (82.5%)	181 (77.0%)	.12
SGLT2i	163 (60.6%)	143 (60.9%)	.95
Loop diuretics	151 (56.1%)	107 (45.5%)	.02
Furosemide equivalent per day, mg	40 [20–60]	40 [20–40]	.10
Thiazides	5 (1.9%)	8 (3.4%)	.28
Digoxin	38 (14.1%)	24 (10.2%)	.18
GDMT score^b^	7.0 [6.0–8.0]	7.0 [5.0–8.3]	.81
ICD	94 (34.9%)	78 (33.2%)	.68
CRT	63 (23.4%)	57 (24.3%)	.83
**Medical history**			
Atrial fibrillation or flutter	128 (47.6%)	123 (52.3%)	.29
COPD	31 (11.5%)	34 (14.5%)	.33
DM	53 (19.7%)	58 (24.7%)	.18
Hypertension	125 (46.5%)	125 (53.2%)	.13
Currently smoking	27 (10.0%)	27 (11.5%)	.60
BMI, kg/m^2^	28.1 ± 5.0	28.3 ± 4.7	.75
**Laboratory results**			
Haemoglobin, mmol/l	9.0 ± 1.0	9.0 ± 1.0	.56
Sodium, mmol/l	139.6 ± 2.5	139.7 ± 2.5	.70
Potassium, mmol/l	4.5 ± 0.4	4.6 ± 0.4	.046
BUN, mmol/l	8.1 ± 3.7	7.9 ± 2.9	.50
eGFR, ml/min/1.73 m^2^	62.8 ± 17.7	61.8 ± 16.8	.51
NT-proBNP, ng/l	450.0 [202.5–1179.8]	461.0 [163.3–1196.0]	.46
Calculated osmolality^c^, mOsm/kg	293.3 ± 5.8	293.7 ± 5.7	.41

Values are *N* (%), mean ± SD or median [interquartile range].

ACEi, angiotensin-converting enzyme inhibitor; ARB, angiotensin receptor blocker; ARNI, angiotensin receptor/neprilysin inhibitor; BMI, body mass index; BUN, blood urea nitrogen; COPD, chronic obstructive pulmonary disease; CRT, cardiac resynchronization therapy; DM, diabetes mellitus; eGFR, estimated glomerular filtration rate; EQ-5D-5L, European Quality of Life Five Dimensions Five Levels questionnaire; FR, fluid restriction; GDMT, guideline-directed medical therapy; HF, heart failure; HFrEF, HF with reduced ejection fraction; HFmrEF, HF with mildly reduced ejection fraction; HFpEF, HF with preserved ejection fraction; ICD, implantable cardioverter–defibrillator; KCCQ-OSS, Kansas City Cardiomyopathy Questionnaire Overall Summary Score; MRA, mineralocorticoid receptor antagonists; NT-proBNP, N-terminal pro–B-type natriuretic peptide; NYHA, New York Heart Association; RAASi, renin–angiotensin–aldosterone system inhibitors; TDS-HF, Thirst Distress Scale for patients with HF; SGLT2i, sodium-glucose cotransporter-2 inhibitor.

^a^Self-reported.

^b^Calculated only for HFrEF, HFmrEF, and HFimpEF patients.

^c^Calculated as [sodium concentration]*2 + [BUN concentration] + [glucose concentration].

### Health status

There was no significant interaction for pre-trial fluid management regarding the KCCQ-OSS (*P*_interaction_ = .43). However, KCCQ-OSS adjusted for baseline values after 3 months was significantly different between randomization arms stratified by pre-trial fluid management (*P* = .035) (*Table 2*).

**Table 2:** Clinical outcomes according to pre-trial fluid management and randomization

	FR to LFI (*N* = 124)	LFI to LFI (*N* = 118)	FR to FR (*N* = 128)	LFI to FR (*N* = 105)	*P* value
**Primary outcome**					
KCCQ-OSS	74.7 (71.2–78.3)	73.2 (69.6–76.9)	73.8 (70.4–77.1)	70.2 (66.3–74.2)	.035^a^
Δ Baseline—Month 3	+1.44 (−1.10–3.97)	−0.62 (−2.98–1.74)	−0.51 (−2.48–1.46)	−3.37 (−5.68–−1.06)	
**Key secondary outcome**					
TDS-HF	17.1 (15.6–18.5)	16.7 (15.1–18.3)	18.4 (17.0–19.9)	18.7 (17.1–20.3)	.019^a^
Δ Baseline—Month 3	−0.78 (−1.96–0.40)	−0.45 (−2.02–1.11)	+1.16 (0.13–2.18)	+2.96 (1.53–4.39)	
**Other secondary outcomes**					
KCCQ-CSS	76.4 (72.9–79.9)	75.4 (71.7–79.1)	76.1 (72.7–79.6)	72.5 (68.6–76.5)	.12^a^
KCCQ-TSS	79.3 (75.7–82.8)	77.7 (73.8–81.5)	79.8 (76.3–83.4)	74.0 (69.8–78.2)	<.001^a^
KCCQ-OSS (−5 to +5)	55 (44.4%)	46 (39.0%)	56 (43.8%)	40 (38.1%)	.084
KCCQ-OSS (−5 or less)	30 (24.2%)	35 (29.7%)	34 (26.6%)	44 (41.9%)	
KCCQ-OSS (+5 or more)	39 (31.5%)	37 (31.4%)	38 (29.7%)	21 (20.0%)	
EQ-5D-5L	0.84 [0.74–0.92]	0.82 [0.70–0.89]	0.81 [0.70–0.91]	0.81 [0.70–0.89]	.70^a^

Clinical outcomes were assessed after 3 months. Values are *N* (%), unadjusted mean (95% confidence interval), or unadjusted median [interquartile range].

EQ-5D-5L, European Quality of Life Five Dimensions Five Levels questionnaire; FR, fluid restriction; HF, heart failure; KCCQ, Kansas City Cardiomyopathy Questionnaire; KCCQ-CSS, KCCQ Clinical Summary Score; KCCQ-OSS, KCCQ Overall Summary Score; KCCQ-TSS, KCCQ Total Symptom Score; LFI, liberal fluid intake; TDS-HF, Thirst Distress Scale for patients with HF.

^a^Based on the adjusted mean difference tested with analysis of covariance using baseline KCCQ value and significant baseline differences (age, sex, β-blockers, loop diuretics, and potassium) as covariates.

Patients who changed from pre-trial FR to LFI had a mean KCCQ-OSS of 74.7 [95% confidence interval (CI) 71.2–78.3] at follow-up, with an increase of 1.44 points (95% CI −1.10–3.97) compared to baseline. In contrast, patients who changed from pre-trial LFI to FR KCCQ-OSS was 70.2 (95% CI 66.3–74.2) after 3 months and was decreased by 3.37 points (95% CI −5.68–−1.06) relative to baseline (*Table 2*).

For patients with pre-trial FR who were randomized to FR, and for patients with pre-trial LFI who were randomized to LFI, KCCQ-OSS after 3 months were 73.8 (95% CI 70.4–77.1) and 73.2 (95% CI 69.6–76.9), respectively. Both groups showed marginal changes between baseline and follow-up scores (*Table 2*).

For other KCCQ subdomains, similar trends were observed: with the highest scores in patients who changed from pre-trial FR to LFI and the lowest scores in patients who changed the other way around [i.e. Clinical Summary Score (*P* = .12), Total Symptom Score (*P* = .041), Symptom Burden Score (*P* = .014), Symptom Frequency Score (*P* = .20), Physical Limitation Score (*P* = .74), Quality of Life (*P* = .067), and Socal Limitation Score (*P* = .043)]. Full results for the KCCQ subdomains are displayed in Supplementary Table S1.

### Thirst distress

No significant interaction was found for pre-trial fluid management and the key secondary outcome TDS-HF (*P*_interaction_ = .42). Nevertheless, after stratification according to pre-trial fluid management, patients randomized to FR had higher thirst distress scores after adjustment for baseline values (*P* = .019) (*Table 2*). The group that changed from a liberal to a restricted fluid intake showed the greatest change in TDS-HF, with an increase of 2.96 (95% CI 1.53–4.39) points from baseline to follow-up at 3 months (*Table 2*).

### Fluid intake

The median patient-reported fluid intake at Week 6 was significantly higher in patients randomized to LFI. Importantly, there was no difference in reported fluid intake between those who adhered to FR or liberal fluid intake prior to study participation (1771 ml [1500–2164] vs 1757 ml [1450–2159], respectively, *P* = .83) (*Table 3*). The proportion of patients with an intake of 2000 to 2500 and 2500 ml or more did not differ between the two groups as well (29 [24.0%] vs 30 [25.2%], and 10 [8.3%] vs 13 [10.9%], respectively, [*P* = .33]). Likewise, no difference in reported fluid intake was observed in patients randomized to FR stratified according to pre-trial fluid management (pre-trial FR: 1486 ml [1408–1554] vs pre-trial liberal intake: 1464 ml [1329–1566], *P* = .39) (*Table 3*).

**Table 3:** Patient-reported fluid intake at Week 6 according to pre-trial fluid management and randomization

	FR to LFI (*N* = 121)	LFI to LFI (*N* = 119)	*P*-value	FR to FR (*N* = 127)	LFI to FR (*N* = 103)	*P*-value
**Reported fluid intake**						
ml	1771 [1500–2164]	1757 [1450–2159]	.83	1486 [1408–1554]	1464 [1329–1566]	.39
ml/kg	21.3 [17.6–25.4]	22.2 [17.1–27.0]	.82	18.1 [15.8–20.7]	17.7 [14.2–20.1]	.23
**Mean fluid intake**			.33			.07
<1500 ml	29 (24.0%)	33 (27.7%)		70 (55.1%)	62 (60.2%)	
1500–1600 ml	17 (14.0%)	5 (4.2%)		34 (26.8%)	17 (16.5%)	
1600–1700 ml	9 (7.4%)	13 (10.9%)		11 (8.7%)	4 (3.9%)	
1700–1800 ml	11 (9.1%)	11 (9.2%)		6 (4.7%)	8 (7.8%)	
1800–1900 ml	8 (6.6%)	7 (5.9%)		1 (0.8%)	1 (1.0%)	
1900–2000 ml	8 (6.6%)	7 (5.9%)		3 (2.4%)	2 (1.9%)	
2000–2500 ml	29 (24.0%)	30 (25.2%)		1 (0.8%)	8 (7.8%)	
≥2500 ml	10 (8.3%)	13 (10.9%)		1 (0.8%)	1 (1.0%)	

Patient-reported fluid intake at Week 6. Values are *N* (%) and median [interquartile range].

FR, fluid restriction; LFI, liberal fluid intake.

### Safety outcomes

No significant differences were observed between groups in (the composite of) death, HF and all-cause hospitalizations or intravenous loop diuretic, or changes in N-terminal pro–B-type natriuretic peptide concentrations or weight (*Table 4*). No differences in oral loop diuretics or other pharmacological HF therapy changes (i.e. initiation, increases, decreases, or termination) were observed between groups, except for the observation that in patients remaining on a LFI, oral loop diuretics were not initiated or increased during the trial, while minor changes were observed in the other three groups (Supplementary Table S2).

**Table 4:** Safety outcomes according to pre-trial fluid management and randomization

	FR to LFI (*N* = 130)	LFI to LFI (*N* = 124)	FR to FR (*N* = 139)	LFI to FR (*N* = 110)	*P* value
**Safety events**					
Death	1 (0.8%)	0 (0.0%)	1 (0.7%)	1 (0.9%)	.80
All-cause hospitalisation	9 (6.9%)	11 (8.9%)	10 (7.2%)	5 (4.5%)	.64
Hospitalization for HF	2 (1.5%)	2 (1.6%)	3 (2.2%)	1 (0.9%)	.89
IV loop diuretics usage	2 (1.5%)	3 (2.4%)	5 (3.6%)	2 (1.8%)	.70
Acute kidney injury^a^	2 (1.5%)	1 (0.8%)	3 (2.2%)	1 (0.9%)	.77
Any changes in loop diuretics	28 (21.5%)	18 (14.5%)	21 (15.1%)	22 (20.0%)	.36
Furosemide equivalent per day, mg	40 [20–40]	40 [20–40]	40 [20–80]	40 [20–40]	.41
Composite of death, any hospitalization, and IV loop diuretics	9 (6.9%)	12 (9.7%)	12 (8.6%)	7 (6.4%)	.76
Composite of death, HF hospitalization, and IV loop diuretics	3 (2.3%)	3 (2.4%)	6 (4.3%)	3 (2.7%)	.75
**NT-proBNP, ng/l** ^b^					
Month 3	444.0 [191.1–1200.0]	383.0 [143.0–1184.0]	492.5 [176.8–1478.8]	633.0 [200.6–1400.0]	.40
Δ Baseline—Month 3^d^	−5.2 [−80.6–108.7]	−8.5 [−100.0–96.0]	−6.0 [−100.0–103.5]	+8.5 [−94.8–200.0]	.76
Month 6	535.5 [192.1–1325.4]	487.5 [172.6–1200.0]	472.0 [166.5–1200.0]	592.0 [207.0–1550.0]	.70
Δ Month 3—Month 6^d^	+5.5 [−102.4–128.3]	+16.9 [−48.3–129.8]	0.0 [−100.0–93.3]	−3.0 [−93.0–194.5]	.83
**Weight, kg** ^c^					
Δ Baseline—Month 3^d^	0.0 [−0.4–1.2]	−0.3 [−2.5–1.2]	0.0 [−1.0–1.0]	−0.1 [−2.0–1.3]	.27
Δ Month 3—Month 6^d^	0.0 [−2.0–1.0]	−0.4 [−3.4–0.9]	0.0 [−1.5–1.0]	+0.4 [−1.1–1.7]	.68

Safety was assessed during the 6 month follow-up.

Values are *N* (%), median [interquartile range] or ratio (95% confidence interval).

FR, fluid restriction; HF, heart failure; IV, intravenous; LFI, liberal fluid intake; NT-proBNP, N-terminal pro–B-type natriuretic peptide.

^a^Acute kidney injury is defined as a 50% decline in estimated glomerular filtration rate relative to baseline, or decrease of >30 ml/min/1.73 m^2^ and to a value below 60 ml/min/1.73 m^2^.

^b^Available values at Month 3 (*N* = 104 vs *N* = 111 vs *N* = 126 vs *N* = 120) and Month 6 (*N* = 93 vs *N* = 108 vs *N* = 109 vs *N* = 106).

^c^Available values at Month 3 (*N* = 43 vs *N* = 47 vs *N* = 96 vs *N* = 98) and month 6 (*N* = 20 vs *N* = 18 vs *N* = 46 vs *N* = 40).

^d^Differences between two timepoints within individual patients with complete data.

## Discussion

This subanalysis of the FRESH-UP study evaluates the effect of pre-trial fluid management on the introduction of a LFI vs an advice of FR in patients with chronic HF. No significant interactions were found for pre-trial fluid management in relation to the primary outcome KCCQ-OSS or the key secondary outcome thirst distress. In other words, in accordance with the primary analysis, there was no benefit of FR regardless of pre-trial fluid management. Moreover, in those with pre-trial FR, there was a signal of benefit for those who were randomized to LFI, whereas for those with pre-trial liberal intake, a signal of harm was demonstrated for FR. Lastly, fluid intake was about 1800 ml per day in those randomized to liberal intake, regardless of pre-trial fluid management. These more in-depth results add insights to the main analysis and warrant further discussion with potentially direct implications for daily clinical practice.^1^

Firstly, patients who changed from pre-trial LFI to FR had the lowest health status and highest thirst distress at follow-up. The highest health status and lowest thirst distress were observed in those with a change in the opposite direction (i.e. from pre-trial FR randomized to an advice of LFI). The first lesson to be learned is that the difference in the primary endpoint analysis of the FRESH-UP study (a nonsignificant mean difference of 2.17 points (*P* = .06) on the KCCQ-OSS in favour of LFI) was driven primarily by the 3.37-point decrease in KCCQ-OSS in patients with a pre-trial liberal fluid intake who were randomized to FR.^1^ These patients also experienced the greatest increase in thirst distress, while in those who changed from FR to LFI, favourable but modest differences were observed in both health status and thirst distress. The lower KCCQ and higher TDS-HF scores observed in the FR group may reflect either a true deterioration in HF, leading to reduced health status and increased thirst distress, or a psychological response to the perception of being restricted. Both mechanisms are clinically relevant and undesirable.

These results mirror those of the only previous study with data on change in fluid management in HF by Holst *et al.*, where thirst sensation was lower during a more liberal intake regimen, while no differences were observed in quality of life, body weight, or use of medication.^3^ Both the study by Holst *et al.* as well as the FRESH-UP study demonstrated no differences in safety events; yet both were obviously not powered for events.^1,3^ However, given the strong predictive value of KCCQ-OSS for clinically important outcomes such as cardiovascular death and hospitalization, it could be hypothesized that there is a signal of harm for FR, while there is not for LFI.^7,8^

A second lesson to be learned from this subanalysis is that daily fluid intake was about 280 ml higher in patients randomized to LFI, and importantly that this difference was independent of pre-trial fluid management. Notably, the mean daily fluid intake was 2000 ml or higher for almost one-third of patients in the liberal group, but the median patient-reported fluid intake in patients randomized to LFI was quite modest at about 1780 ml. One may put forward that fluid intake might have been modest due to previous FR advice given to the patient. This is however contradicted by the results of the current subanalysis, which demonstrates that fluid intake was not impacted by previous fluid management. As shown, patients with pre-trial LFI who were randomized to continue LFI also drank about 1780 ml per day. It is likely that the reported intake of 1780 ml per day during the trial also reflects their intake prior to the trial for an extended period of time. At first, these results raise the question why HF patients with LFI seem to have such modest intake. But importantly, 1780 ml of daily fluid intake actually reflects a normal daily fluid intake for HF patients with a mean age of 70 years and is similar to those of the same age in the general population and enough for most patients to quench their thirst.^9–11^ This population may not need nor want to consume more or less than that.

There are other observations to support this hypothesis. In the study by Holst *et al.*, 74 HF patients were randomized to a strict FR of 1500 ml/day or a more liberal weight-based fluid advice of 30 ml/kg/day.^3^ Importantly, during the more liberal study period, patients actually only drank on average 23 ml/kg/day, far below the advised limit of intake. Moreover, in a study by Travers *et al.*, 67 patients with acute HF (all NYHA IV and mean age 73 years), patients were randomized to LFI or FR.^4^ The mean daily intake of patients randomized to LFI was only 1466 ± 607 ml. In addition, in patients with stage 3 chronic kidney disease without HF, an advice to increase normal daily intake by an additional 1000 ml of water only resulted in an increase of 600 ml per day.^12^ Also, patients suffering from headaches were only able to increase their fluid intake by 800 ml instead of the advised 1500 ml.^13^ In summary, these studies suggest that people in general do not want to drink more than normal volumes of 1500–2500 ml/day.^14^

The lack of any signal of harm related to LFI supports the notion that signs and symptoms of fluid retention are not determined by fluid intake.^14,15^ It is important to clarify that ingested fluid will disperse equally over all fluid compartments of the human body. For example, 1000 ml of fluids results in only 120 ml extra to the intravascular compartment, while the splanchnic venous system is able to mobilize 800 ml within seconds, and the kidneys are able to clear 1000 ml of free water within 2.5 hours completely.^14–16^ In this light, also an intake well above 1780 ml per day is unlikely to adversely affect clinical outcomes in HF, especially in those with relatively stable disease with high uptake of guideline-directed medical therapy.^14–16^

When these exploratory findings are considered in a clinical context, it is possible that, for patients who currently adhere to LFI, a change to an advice of FR may result in lower health status and more thirst distress. For patients with a current FR, an advice of LFI may result in less thirst distress and no detrimental effect on health status or clinical outcomes. This would be in accordance with the recent consensus statement of the HF association of the ESC, which recommends a normal fluid intake between 1500 and 2500 ml in patients with chronic HF, and that even higher amounts may be allowed while there seems no need to provoke a certain amount of fluid intake.^14^

Importantly, the study population comprised predominantly white male patients with relatively stable chronic HF in NYHA class II or III, a sodium concentration of ≥130 mmol/l and a renal function of ≥30 ml/min/1.73 m^2^, which limits generalizability to the HF population as a whole, especially to those with more advanced and acute HF.

### Limitations

In addition to the above, the FRESH-UP study has other limitations, which have been discussed previously (these include the relatively small population, short duration of follow-up, the way the lifestyle advice was presented to the participants may have influenced the outcome measures, and the interpretation of the TDS-HF requires caution as it has not yet been correlated with quality of life).^1^ Due to the open-label design of the study, reporting bias cannot be excluded. Also, the study was not powered for hard clinical endpoints or this subanalysis. In addition, although the concept of the current subgroup analysis was prespecified in the statistical analysis plan, it was adapted post-hoc. Any subanalysis (including the current subanalysis with nonsignificant interaction) should be interpreted as exploratory only. Importantly, information on actual pre-trial fluid intake instead of self-reported pre-trial fluid management could have added insight to the current analysis, but these data were not assessed. Lastly, a potential influence of the timing of the fluid intake diary cannot be fully excluded.

## Conclusions

This subanalysis exposes a signal of potential harm of FR in patients with pre-trial LFI and a potential benefit for a change from FR to a LFI. The daily fluid intake was about 280 ml higher in patients randomized to LFI, which was not affected by pre-trial fluid management. These results add to understanding the overall results of the FRESH-UP study and support that there is no need to routinely initiate or continue FR in stable patients with chronic HF.

## Supplementary Material

xvag197_Supplementary_Data

## Declarations

### Data Availability

No data were generated or analysed for this manuscript.

### Pre-registered Clinical Trial Number

**Trial Registration:** NCT04551729.

## References

[1] Herrmann JJ, Brunner-La Rocca H-P, Baltussen LEHJM, Beckers-Wesche F, Bekkers SCAM, Bellersen L, et al Liberal fluid intake versus fluid restriction in chronic heart failure: a randomized clinical trial. Nat Med 2025;31:2062–8. 10.1038/s41591-025-03628-4 40159556

[2] Albert NM, Nutter B, Forney J, Slifcak E, Tang WH. A randomized controlled pilot study of outcomes of strict allowance of fluid therapy in hyponatremic heart failure (SALT-HF). J Card Fail 2013;19:1–9. 10.1016/j.cardfail.2012.11.007 23273588

[3] Holst M, Stromberg A, Lindholm M, Willenheimer R. Liberal versus restricted fluid prescription in stabilised patients with chronic heart failure: result of a randomised cross-over study of the effects on health-related quality of life, physical capacity, thirst and morbidity. Scand Cardiovasc J 2008;42:316–22. 10.1080/14017430802071200 18609051

[4] Travers B, O'Loughlin C, Murphy NF, Ryder M, Conlon C, Ledwidge M, et al Fluid restriction in the management of decompensated heart failure: no impact on time to clinical stability. J Card Fail 2007;13:128–32. 10.1016/j.cardfail.2006.10.012 17395053

[5] Herrmann JJ, van Berlo R, Brunner-La Rocca H-P, Sanders-Van Wijk S, Gommans DHF, van Kimmenade RRJ. Fluid restriction in patients with heart failure: a systematic review. Heart 2026. 10.1136/heartjnl-2025-326784 41529956

[6] Herrmann JJ, Beckers-Wesche F, Baltussen LEHJM, Verdijk MHI, Bellersen L, Brunner-la Rocca H-P, et al Fluid REStriction in heart failure vs liberal fluid UPtake: rationale and design of the randomized FRESH-UP study. J Card Fail 2022;28:1522–30. 10.1016/j.cardfail.2022.05.015 35705150

[7] Spertus JA, Jones PG, Sandhu AT, Arnold SV. Interpreting the Kansas city cardiomyopathy questionnaire in clinical trials and clinical care: JACC state-of-the-art review. J Am Coll Cardiol 2020;76:2379–90. 10.1016/j.jacc.2020.09.542 33183512

[8] Abdel Jawad M, Jones PG, Arnold SV, Cohen DJ, Sherrod CF, Khan MS, et al Interpreting population mean treatment effects in the Kansas city cardiomyopathy questionnaire: a patient-level meta-analysis. JAMA Cardiol 2025;10:32–40. 10.1001/jamacardio.2024.4470 39546393 PMC11841199

[9] Masot O, Miranda J, Santamaria AL, Paraiso Pueyo E, Pascual A, Botigué T. Fluid intake recommendation considering the physiological adaptations of adults over 65 years: a critical review. Nutrients 2020;12:3383. 10.3390/nu12113383 33158071 PMC7694182

[10] Knowledge4Policy . Intake of drinking water and total non-alcoholic beverage intake. https://knowledge4policy.ec.europa.eu/health-promotion-knowledge-gateway/defining-water-overview-4_en (15 April, 2025, date last accessed)

[11] van Rossum CT, Sanderman-Nawijn EL, Brants HA, Dinnissen CS, Jansen-Van der Vliet M, Beukers MH, et al The Diet of the Dutch. Results of the Dutch National Food Consumption Survey 2019-2021 on Food Consumption and Evaluation with Dietary Guidelines. Bilthoven, The Netherlands: RIVM, 2023.

[12] Clark WF, Sontrop JM, Huang S-H, Gallo K, Moist L, House AA, et al Effect of coaching to increase water intake on kidney function decline in adults with chronic kidney disease: the CKD WIT randomized clinical trial. JAMA 2018;319:1870–9. 10.1001/jama.2018.4930 29801012 PMC6583759

[13] Spigt M, Weerkamp N, Troost J, van Schayck CP, Knottnerus JA. A randomized trial on the effects of regular water intake in patients with recurrent headaches. Fam Pract 2012;29:370–5. 10.1093/fampra/cmr112 22113647

[14] Mullens W, Damman K, Dhont S, Banerjee D, Bayes-Genis A, Cannata A, et al Dietary sodium and fluid intake in heart failure. A clinical consensus statement of the Heart Failure Association of the ESC. Eur J Heart Fail 2024;26:730–41. 10.1002/ejhf.3244 38606657

[15] Fallick C, Sobotka PA, Dunlap ME. Sympathetically mediated changes in capacitance: redistribution of the venous reservoir as a cause of decompensation. Circ Heart Fail 2011;4:669–75. 10.1161/CIRCHEARTFAILURE.111.961789 21934091

[16] Levick JR . Capillary filtration-absorption balance reconsidered in light of dynamic extravascular factors. Exp Physiol 1991;76:825–57. 10.1113/expphysiol.1991.sp003549 1768414

