# Supplementary material for: The effect of a change in fluid intake advice on outcomes in chronic heart failure: a prespecified subanalysis of the FRESH-UP study
Source: ESC Heart Fail. 2026 Jul 29;13(5):xvag197. doi: 10.1093/eschf/xvag197 (PMC13557008; doi:10.1093/eschf/xvag197)
Supplement: Supplementary file 1 [file xvag197_supplementary_data.docx]

# The Effect of a Change in Fluid Intake Advice on Outcomes in Chronic Heart Failure. Supplementary appendix.

## Table of Content.

Table S1: KCCQ domains according to pre-trial fluid management and randomization

Table S2: Medication changes

# Table S1: KCCQ domains according to pre-trial fluid management and randomization

|  | **FR to LFI**  **(N = 124)** | **LFI to LFI**  **(N = 118)** | **FR to FR**  **(N = 128)** | **LFI to FR**  **(N = 105)** | **P value** |
| --- | --- | --- | --- | --- | --- |
| **KCCQ-Overall Summary Score** | 74.7 (71.2 – 78.3) | 73.2 (69.6 – 76.9) | 73.8 (70.4 – 77.1) | 70.2 (66.3 – 74.2) | 0.035^†^ |
| Δ Baseline – Month 3 | +1.44 (-1.10 – 3.97) | -0.62 (-2.98 – 1.74) | -0.51 (-2.48 – 1.46) | -3.37 (-5.68 – -1.06) |  |
| **KCCQ-Clinical Summary Score** | 76.4 (72.9 – 79.9) | 75.4 (71.7 – 79.1) | 76.1 (72.7 – 79.6) | 72.5 (68.6 – 76.5) | 0.12^†^ |
| Δ Baseline – Month 3 | +1.81 (-0.71 – 4.33) | +1.06 (-1.29 – 3.41) | -0.28 (-2.35 – 1.79) | -2.26 (-4.42 - -0.10) |  |
| **KCCQ-Total Symptom Score** | 79.3 (75.7 – 82.8) | 77.7 (73.8 – 81.5) | 79.8 (76.3 – 83.4) | 74.0 (69.8 – 78.2) | 0.041^†^ |
| Δ Baseline – Month 3 | +2.62 (-0.37 – 5.61) | +2.32 (-0.76 – 5.40) | -0.26 (-3.18 – 2.66) | -3.40 (-6.32 – -0.48) |  |
| **KCCQ-Symptom Burden Score** | 78.3 (74.5 – 82.1) | 77.1 (73.0 – 81.3) | 80.5 (76.7 – 84.2) | 72.5 (67.9 – 77.0) | 0.014^†^ |
| Δ Baseline – Month 3 | +2.15 (-1.42 – 5.72) | +3.25 (-0.33 – 6.83) | +0.78 (-2.71 – 4.27) | -4.05 (-7.68 – -0.41) |  |
| **KCCQ-Symptom Frequency Score** | 80.3 (76.6 – 83.9) | 78.2 (74.4 – 82.1) | 77.2 (75.5 – 82.9) | 75.6 (71.3 – 79.8) | 0.20^†^ |
| Δ Baseline – Month 3 | +3.09 (0.02 – 6.16) | +1.39 (-1.76 – 4.55) | -1.30 (-4.17 – 1.57) | -2.76 (-5.64 – 0.12) |  |
| **KCCQ-Physical Limitation Score** | 73.0 (69.1 – 77.0) | 72.9 (68.8 – 77.0) | 72.3 (68.3 – 76.2) | 71.1 (66.7 – 75.5) | 0.74^†^ |
| Δ Baseline – Month 3 | +0.92 (-1.83 – 3.66) | -0.39 (-2.96 – 2.17) | -0.07 (-2.50 – 2.37) | -1.11 (-3.87 – 1.64) |  |
| **KCCQ-Quality of Life Score** | 73.9 (69.8 – 77.9) | 71.8 (67.8 – 75.7) | 72.7 (69.0 – 76.3) | 69.1 (64.5 – 73.7) | 0.067^†^ |
| Δ Baseline – Month 3 | +0.67 (-2.57 – 3.91) | -0.78 (-4.09 – 2.54) | -0.39 (-3.37 – 2.59) | -4.64 (-7.94 – -1.34) |  |
| **KCCQ-Social Limitation Score** | 71.9 (67.5 – 76.3) | 70.1 (65.3 – 74.8) | 69.8 (65.4 – 74.3) | 66.5 (61.6 – 71.5) | 0.043^†^ |
| Δ Baseline – Month 3 | +0.70 (-2.78 – 4.18) | -5.05 (-8.60 – -1.51) | -1.50 (-4.47 – 1.47) | -3.77 (-7.76 – 0.22) |  |
| **KCCQ-Self-Efficacy Score** | 86.3 (83.0 – 89.6) | 80.0 (76.3 – 83.7) | 86.6 (83.9 – 89.4) | 82.6 (78.7 – 86.5) | 0.11^†^ |
| Δ Baseline – Month 3 | +1.41 (-2.31 – 5.13) | +1.27 (-3.04 – 5.58) | -0.29 (-3.20 – 2.62) | +0.83 (-3.29 – 4.96) |  |
| **KCCQ-Symptom Stability Score** | 50.4 (46.8 – 54.0) | 50.4 (47.6 – 53.2) | 52.3 (49.8 – 54.9) | 49.5 (46.6 – 52.4) | 0.47^†^ |
| Δ Baseline – Month 3 | -4.03 (-8.65 – 0.58) | -0.85 (-4.27 – 2.57) | -2.93 (-6.64 – 0.78) | -2.86 (-6.61 – 0.90) |  |

**Legend**

KCCQ subdomains after 3 months.
Total Symptom Score = mean of Symptom Burden Score and Symptom Frequency Score.
Clinical Summary Score = mean of Total Symptom Score and Physical Limitation Score.
Overall Summary Score = mean of Total Symptom Score, Physical Limitation Score, Quality of Life Score and Self Limitation Score.
Values are unadjusted mean (95% confidence interval).

FR = Fluid Restriction; KCCQ = Kansas City Cardiomyopathy Questionnaire; LFI = Liberal Fluid Intake.
† Based on the adjusted mean difference tested two-sided with ANCOVA analysis using baseline KCCQ value and significant baseline differences (age, sex, beta-blockers, loop diuretics and potassium) as covariate.

# Table S2: Medication changes

|  | **FR to LFI**  **(N = 130)** | **LFI to LFI**  **(N = 124)** | **FR to FR**  **(N = 139)** | **LFI to FR**  **(N = 110)** | **P value** |
| --- | --- | --- | --- | --- | --- |
| **Between baseline and month 3** |  |  |  |  |  |
| Loop diuretics any changes | 16 (12.3%) | 5 (4.0%) | 13 (9.4%) | 10 (9.1%) | 0.13 |
| Loop diuretics dose increased or initiation | 10 (7.7%) | 0 (0.0%) | 8 (5.8%) | 6 (5.5%) | 0.028 |
| Loop diuretics dose decreased or termination | 6 (4.6%) | 5 (4.0%) | 5 (3.6%) | 4 (3.6%) | 0.97 |
| Any HF medication changes ^†^ | 30 (23.1%) | 18 (14.5%) | 26 (18.7%) | 23 (20.9%) | 0.36 |
| **Between month 3 and month 6** |  |  |  |  |  |
| Any loop diuretics changes | 14 (10.8%) | 14 (11.3%) | 12 (8.6%) | 15 (13.6%) | 0.66 |
| Loop diuretics dose increased or initiation | 7 (5.4%) | 12 (9.7%) | 8 (5.8%) | 11 (10.0%) | 0.35 |
| Loop diuretics dose decreased or termination | 7 (5.4%) | 4 (3.2%) | 7 (5.0%) | 6 (5.5%) | 0.83 |
| Any HF medication changes ^†^ | 31 (23.8%) | 36 (29.0%) | 24 (17.3%) | 29 (26.4%) | 0.14 |
| **During study follow-up** |  |  |  |  |  |
| Loop diuretics any changes | 28 (21.5%) | 18 (14.5%) | 21 (15.1%) | 22 (20.0%) | 0.36 |
| Loop diuretics dose increased or initiation | 17 (13.1%) | 12 (9.7%) | 15 (10.8%) | 14 (12.7%) | 0.81 |
| Loop diuretics dose decreased or termination | 12 (9.2%) | 8 (6.5%) | 12 (8.6%) | 10 (9.1%) | 0.85 |
| Any HF medication changes^†^ | 50 (38.5%) | 48 (38.7%) | 40 (28.8%) | 42 (38.2%) | 0.25 |

**Legend**

Values are N (%).

FR = Fluid Restriction; HF = Heart Failure; LFI = Liberal Fluid Intake.

† Heart failure medication changes comprises changes in renin–angiotensin–aldosterone system inhibitors, beta-blockers, mineralocorticoid receptor antagonists, sodium-glucose cotransporter-2 inhibitors, loop and thiazide diuretics.
